# Die Färbetechnik des Nervensystems

**Published:** 1898-03

**Authors:** 


**Die Färbetechnik des Nervensystems.** Von Dr. B. Pollack. Pp. v., 130. Berlin: S. Karger. 1897.

This will be found a very valuable little book to those who are working at the pathology of the nervous system. It gives an account of the various methods employed in the preparation of macroscopical and microscopical sections of the brain and spinal cord. As many of these methods are extremely complicated, and the description of some of them has to be hunted out in various journals, it is exceedingly convenient to have them collected together in the handy form of this volume. The author has dealt with his subject in a very complete and satisfactory manner, the description of the various processes being clearly put, and the most recent methods being included. He presupposes on the part of the reader some general knowledge of the elementary processes used in the cutting and preparing of microscopical sections. The author has compiled a most useful book, which will be found indispensable to all workers at this subject. It forms a volume of convenient size, which is clearly printed, well arranged for reference, with a sufficient index. There are some useful general directions at the end of the book.

